# Dose-Related Aberrant Inhibition of Intracellular Perforin Expression by S1 Subunit of Spike Glycoprotein That Contains Receptor-Binding Domain from SARS-CoV-2

**DOI:** 10.3390/microorganisms9061303

**Published:** 2021-06-15

**Authors:** Chun-Fu Huang, Szu-Min Hsieh, Sung-Ching Pan, Yu-Shang Huang, Shan-Chwen Chang

**Affiliations:** Division of Infectious Diseases, Department of Internal Medicine, National Taiwan University Hospital and College of Medicine, National Taiwan University, Taipei 100, Taiwan; dkseneren@gmail.com (C.-F.H.); scpbpan@gmail.com (S.-C.P.); b101091021@gmail.com (Y.-S.H.); changsc@ntu.edu.tw (S.-C.C.)

**Keywords:** Covid-19, SARS-CoV-2, perforin, cytokine storm syndrome, perforin, lymphoproliferation, cytotoxicity

## Abstract

Studies had shown that severe cases of COVID-19 tend to have high viral loads and correlate with functional impairment of cytotoxic lymphocytes, and the features of cytokine storm syndrome are similar to manifestations of severe influenza that have been partially explained by suppressed perforin expression. To test the hypothesis that the spike glycoprotein from SARS-CoV-2 may inhibit the perforin expression, we determined the kinetics of immune responses of CD8+ T cells to low dose (LD) or high dose (HD) of S1 stimulation through an in vitro dendritic cell (DC)-T cell model over seven days of incubation. The cytotoxic activity and intracellular perforin expression of CD8+ T cells induced by HD-S1-presenting DCs were aberrantly lower than those induced by LD-S1-presenting DCs from day three of incubation. Discrepantly, the levels of lymphoproliferation and cytokine (interferon-γ and tumor necrosis factor-α) production induced by HD-S1-presenting DCs were significantly higher than those induced by LD-S1-presenting DCs from day four. The dose-related responses between doses of S1 and intracellular perforin expression showed a significant linear correlation with a negative slope. In conclusion, the S1 subunit may suppress the perforin expression in CD8+ T cells to decrease the cytotoxic capacity to kill spike-presenting cells in a dose-dependent manner; the persistence of antigen presentation may result in an overproduction of interferon-γ and subsequent proinflammatory cytokines. That may help explain the insufficient cytotoxicity against high quantities of viruses or highly replicated strains of SARS-CoV-2 in severe cases of COVID-19.

## 1. Introduction

Coronavirus disease 2019 (COVID-19) emerged in Wuhan, China, in December 2019, which is caused by severe acute respiratory syndrome coronavirus 2 (SARS-CoV-2) [1]. Respiratory failure from acute respiratory distress syndrome (ARDS) is the leading cause of mortality in severe cases [2]. Immune-mediated pulmonary injury or ARDS in Covid-19 results from dysregulated hypercytokinemia, the conditions called cytokine storm syndrome (CSS), secondary hemophagocytic lymphohistiocytosis (sHLH), or macrophage activation syndrome (MAS), characterized by increased plasma levels of interferon (IFN)-γ and proinflammatory cytokines, including interleukin (IL)-2 receptor, IL-6, IL-10, and tumor necrosis factor (TNF)-α [3,4,5]. The phenomenon has been recognized in severe cases of infections by highly pathogenic coronaviruses, including severe acute respiratory syndrome coronavirus (SARS-CoV), SARS-CoV-2, and Middle East respiratory syndrome coronavirus (MERS-CoV) [3,4,5,6,7,8,9], or influenza viruses including H5N1 [10,11,12]. IFN-γ has been considered to play an essential role [13], and the IL-6-related JAK (Janus kinases)-STAT3 (signal transducer and activator of transcription 3) signaling pathway has also been elucidated and supposed to be critically involved [14]. However, how and when SARS-CoV-2 infection initiates the process of cytokine cascade remains unknown.

Perforin is a key component of the lytic granules in CD8+ cytotoxic T-lymphocyte (CTL) [15,16]. Viral infections of perforin-deficient mice or persons with perforin gene defects, such as type 2 familial hemophagocytic lymphohistiocytosis (FHL 2), may result in the characteristic presentations of CSS when viral infection [17,18,19]. In addition, a recent study had shown that the viral loads of severe cases of COVID-19 were much higher than those of mild cases, suggesting that higher viral loads might be associated with severe clinical outcomes [20]. Furthermore, there was evidence to show that the progression of COVID-19 to severe disease correlates with functional impairment of CTL in vivo [21]. According to the above findings from the literature, we hypothesized that the cytokine cascade in CSS might be initiated through the ineffective CTL activity that is secondary to insufficient perforin expression if patients are infected with high quantities of viruses or highly replicated viral strains of SARS-CoV-2.

The risk and benefit of steroid use in severe cases of COVID-19 remains a highly controversial issue [6,22,23,24,25]. If the CSS is the result of insufficient CTL that fails to clear the virus, the overuse of steroids or other immunosuppressants in CSS may worsen the host defense against SARS-CoV-2 and may predict a poor outcome as in cases of SARS, MERS, and influenza [26,27,28]. In this study, we determined and compared the kinetics of immune responses in CD8+ T cells in response to high dose or low dose of recombinant S1 subunit of spike glycoprotein, prepared from SARS-CoV-2 or from low pathogenic coronavirus (HCoV-HKU1), to assess the possible effects of the receptor-binding domain (RBD)-containing spike glycoprotein on the suppression of intracellular perforin expression in CD8+ T cells and the possible dose-related responses.

## 2. Methods

### 2.1. Preparation of Antigen-Presenting Monocyte-Derived Dendritic Cells (DCs)

To mimic the in vivo condition of antigen presentation and T cell recognition and to carefully control the effector/target cell ratios, we applied an in vitro model of dendritic cell—T cell interaction (in vitro DC-T model). We prepared monocyte-derived immature DCs as professional antigen-presenting cells (APCs) from the PBMCs isolated from the 10 healthy volunteers aged from 30–48 years old, without detectable antibodies to SARS-CoV-2 (ALERT™ SARS-CoV-2 Antibody Test, EBS, Taipei, Taiwan), and all subjects provided written informed consent. This study was conducted in accordance with Good Clinical Practice, the Declaration of Helsinki, and with the approval of the local ethics committee (27 April 2020, No. 202004027RIND). The methods for preparing antigen-presenting DCs have been described previously [12,29]. Briefly, monocytes were negatively isolated from PBMCs using the immunomagnetic bead method (Dynabead^TM^ Untouch^TM^ Human Monocytes Kit; Invitrogen, Waltham, MA, USA) according to the manufacturer’s instructions. Negatively isolated monocytes were suspended in complete RPMI 1640 (Invitrogen Life Technologies) supplemented with recombinant human IL-4 (1000 U/mL; BD Pharmingen, San Diego, CA, USA) and GM-CSF (50 ng/mL; BD Pharmingen, San Diego, CA, USA) in 24-well plates (1 mL/well) with a cell concentration of 10^6^/mL in triplicate. These monocytes showed the phenotype of immature DCs on Day 6 of cell cultures. We pulsed these immature DCs (10^5^/mL RPMI 1640 in 24-well plates) with purified recombinant spike glycoproteins from SARS-CoV-2 or HCoV-HKU1 (described as below) for another 24 h, and then we co-incubated these antigen-presenting DCs with isolated autologous CD8+ T cells (as described below) with a ratio of CD8+ T cells:DCs of 10:1. The viability of antigen-presenting DCs, before and after incubation with CD8+ T cells was estimated by a counting chamber with characteristic morphology of DCs and trypan blue dye exclusion.

### 2.2. Antigens

The Spike protein of coronavirus is composed of two subunits, S1 and S2. The receptor-binding domain is located in S1. The following recombinant spike glycoproteins from highly pathogenic coronavirus (SARS-CoV-2) were used as antigens in this study (all from Sino Biological, Beijing, China): receptor-binding domain (RBD, Arg319-Phe541, MW 26.5 kDa); RBD-containing S1 subunit (HS1, Val16—Arg685, MW 76.5 kDa); extracellular domain (ECD)-containing S2 subunit (HS2, Ser686—Pro1213, MW 59.4 kDa); full-length spike glycoprotein (FLS, S1 and S2; Val16—Pro1213, MW 134.4 kDa).

We also applied the RBD-containing S1 subunit prepared from low pathogenic coronavirus (LS1, Ala13-Arg756, MW 85.2 kDa, isolate N5, HCoV-HKU1) for comparison. Endotoxin was determined at <1.0 EU per μg protein by the LAL method. In our preliminary experiments, the concentrations of LS1 < 80 ng/mL could not induce reproducible lymphoproliferation activity of CD8+ T cells in our methods; the optimal doses of LS1 to induce reproducible antigen-specific lymphoproliferation activity of CD8+ T cells seemed to be between 100 and 800 ng/mL. Therefore, in this study, we used two doses of LS1: low dose (LD) at 120 ng/mL and high dose (HD) at 600 ng/mL. The doses of other antigens were calculated according to the relative molecular weights to achieve nearly equivalent amounts of protein molecules, such as LD-RBD as 40 ng/mL and HD-RBD as 200 ng/mL, LD-HS1 as 110 ng/mL, and HD-HS1 as 550 ng/mL; LD-HS2 as 80 ng/mL and HD-HS2 as 400 ng/mL; LD-FLS as 200 ng/mL and HD-FLS as 1000 ng/mL, in some experiments.

### 2.3. Cell Isolation

CD8+ T cells were positively isolated from PBMCs and detached from beads by an immunomagnetic method (CD8 Positive Isolation Kit; Dynabeads^®^ plus DETACHaBEAD^®^; Invitrogen) according to the manufacturer’s instructions. The resulting purity was >99%, and the viability was >95%.

### 2.4. Measurement of Antigen-Specific Responses of CD8+ T Cells

The CD8+ T cell responses to purified recombinant antigens were assessed by incubating CD8+ T cells with autologous DCs, which remained at an immature state or already pulsed with antigens for 24 h in a final volume of 1 mL of RPMI 1640 (10^6^ CD8+ T cells with 10^5^ DCs per well of a 24-well plate in triplicate). The CD8+ T cells were then harvested for measurement of cytotoxicity, lymphoproliferation, intracellular perforin expression, and the supernatants were harvested for determination of concentrations of cytokines on the varying duration of incubation.

### 2.5. Antigen-Specific Cytotoxic Response of CD8+ T Cells

Isolated CD8+ T cells (10^6^/well) were prepared as effector cells by incubating with autologous antigen-presenting DCs (as APCs and target cells, with E/T ratio of 10:1) in a final volume of 1 mL of complete RPMI 1640 in 24-well plates with variable duration (from day 0–day 7 of incubation). In this study, a non-radioactive lactate dehydrogenase (LDH)-releasing cytotoxicity assay kit (CytoTox 96 Non-Radioactive Cytotoxicity Assay; Promega) was used to determine the antigen-specific CTL activity according to the manufacturer’s instructions, as described previously [12,29]

### 2.6. Antigen-Specific Fluorescence Intensity of Perforin Expression

**Figure 1:**
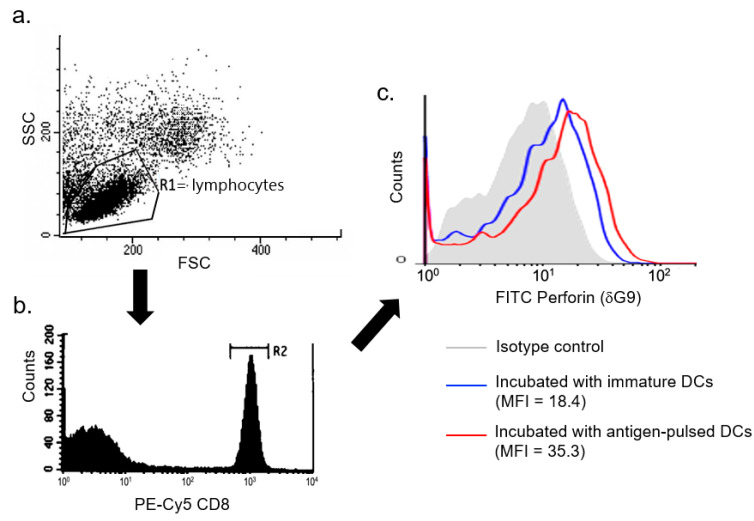
Gating strategy for the determination of mean fluorescence intensity (MFI) of intracellular perforin expression in CD8+ T cells. The figure shown is a representative analysis. Samples were analyzed using the following gating strategy: (**a**) Lymphocyte gating (SSC vs. FSC) → (**b**) CD8+ T cells → (**c**) intracellular perforin expression, and MFI determination only in R2-gated cells to exclude dead cells or droplets.

Intracellular perforin expression of CD8+ T cells was measured on the varying days of DC-T incubation. After cell surface staining with mouse anti-human CD8 (PE-Cy5-conjugated HIT8a, Mouse IgG1κ; BD Pharmingen), cells were washed twice and resuspended in cold Dulbecco’s PBS and then fixed and permeabilized by Cytofix/Cytoperm solution (15 min, 4 °C, in the dark; BD Pharmingen) according to the manufacturer’s protocol. These fixed and permeabilized cells were stained with mAb specific for human perforin (FITC-conjugated δG9, mouse IgG2bκ; BD Pharmingen) or isotype control (20 μL/10^6^ cells) at room temperature for 30 min in the dark, and then analyzed by flow cytometry (the gating strategy as described in Figure 1). The mean fluorescence intensity (MFI) of antigen-specific intracellular perforin expression was defined as the MFI of intracellular perforin expression in CD8+ T cells incubated with antigen-presenting DCs minus the MFI of intracellular perforin expression in CD8+ T cells incubated with immature DCs without being pulsed with antigen.

### 2.7. Antigen-Specific Response of Lymphoproliferation

The lymphoproliferation activity of CD8+ T cells was assessed by determining the frequencies of CD8+ T cells with BrdU incorporation using FITC BrdU Flow Kit (BD Pharmingen^TM^) in triplicate. BrdU (final concentration 10 μM) was added 24 h before harvest. Cells with BrdU incorporation were detected by flow cytometry after being fixed with paraformaldehyde, permeabilized with saponin, and stained with anti-BrdU FITC according to the protocol from the manufacturer. The antigen-specific lymphoproliferation responses of CD8+ T cells were defined as follows: the CD8+ T cell responses elicited by antigen-presenting DCs minus the CD8+ T cell responses elicited by immature DCs without being pulsed with antigen.

### 2.8. Antigen-Specific Production of Cytokines

Supernatants of the cell cultures were harvested on varying days of incubation and frozen at −70 °C until used. Levels of IFN-γ, TNF-α, and IL-6 were determined using a commercial ELISA kit (Quantikine; R&D Systems, Minneapolis, MN, USA) according to the manufacturer’s instructions. The antigen-specific cytokine production was defined as the levels of cytokines in supernatants from cell cultures containing CD8+ T cells and antigen-presenting DCs minus the levels of cytokines in supernatants from cell cultures containing CD8+ T cells and immature DCs without being pulsed with antigen, on the same day of co-incubation.

### 2.9. Statistical Analysis

Statistical significance was determined using a nonparametric test (Wilcoxon signed-rank test) if two paired groups were compared, or repeated-measures one-way ANOVA was used if more than two groups were compared. Linear correlation was evaluated by Pearson’s correlation coefficient. All tests were two-tailed, and *p* < 0.05 was considered statistically significant. All data are shown as mean ± SD.

## 3. Results

### 3.1. Cytotoxicity and Perforin Expression

**Figure 2:**
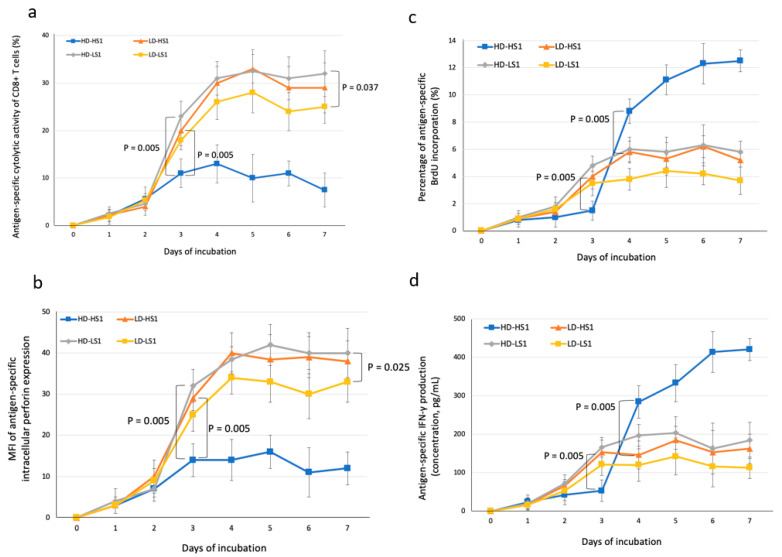
Kinetics of dendritic cells (DC)-elicited spike-specific CD8+ T cell responses to the purified recombinant receptor-binding domain (RBD)-containing S1 subunits (HS1: S1 of highly pathogenic SARS-CoV-2; LS1: S1 of low pathogenic HCoV-HKU1) with nearly equivalent numbers of molecules. (**a**) Cytotoxic activity (assessed by LDH-releasing cytotoxicity assay); (**b**) Mean fluorescein intensity (MFI) of intracellular perforin expression (stained with FITC-conjugated δG9 and assessed by flow cytometry); (**c**) Lymphoproliferation responses (assessed by BrdU incorporation assay); (**d**) IFN-γ production in cell culture supernatants (assessed by ELISA). Data are presented as mean + SD among 10 subjects. High dose (HD) and low dose (LD) of individual antigens as following: LD-LS1 as 120 ng/mL and HD-LS1 as 600 ng/mL; LD-HS1 as 110 ng/mL and HD-HS1 as 550 ng/mL, to achieve the nearly equivalent numbers of protein molecules; *p*-values were determined by Wilcoxon signed-rank test.

We defined the kinetics of antigen-specific responses of CD8+ T cells elicited by autologous monocyte-derived DCs pulsed with low dose (LD) or high dose (HD) of S1 subunit of spike glycoprotein from highly pathogenic coronavirus SARS-CoV-2 (LD-HS1 or HD-HS1) or from low pathogenic coronavirus HCoV-HKU1 (LD-LS1 or HD-LS1) over seven days of co-incubation. The overall pattern of antigen-specific cytotoxic activity of CD8+ T cells was similar to that of antigen-specific intracellular perforin expression in CD8+ T cells (Figure 2a,b). The HD-LS1-specific CD8+ CTL activity and intracellular perforin expression in CD8+ T cells were significantly higher than the LD-LS1-specific responses on day seven (*p* = 0.037 and 0.025, respectively). The LD-HS1-specific CD8+ CTL activity and intracellular perforin expression in CD8+ T cells were similar to the HD-LS1-specific responses (Figure 2a,b). However, the HD-HS1-specific CD8+ CTL activity and intracellular perforin expression in CD8+ T cells were significantly lower than the HD-LS1-specific responses (*p* = 0.005 and 0.005, respectively) and also lower than the LD-HS1-specific responses (*p* = 0.005 and 0.005, respectively) on day three of incubation (Figure 2a,b).

### 3.2. Antigen-Specific Lymphoproliferation and IFN-γ Production

We assessed the kinetics of antigen-specific lymphoproliferation of CD8+ T cells and IFN-γ levels in supernatants in response to autologous antigen-presenting DCs. The results showed the overall kinetics of antigen-specific lymphoproliferation in CD8+ T cells were similar to those of antigen-specific IFN-γ production (Figure 2c,d). The levels of the HD-HS1-specific lymphoproliferation of CD8+ T cells and IFN-γ levels in supernatants showed an initially lower (at ≤3 days of incubation) but significantly higher levels of responses later (at ≥4 days of incubation) than those of the LD-HS1-specific responses. Therefore, a high dose of HS1 was associated with significantly lower levels of intracellular perforin expression and CTL activity of CD8+ T cells (from day three) but with significantly higher levels of lymphoproliferation and IFN-γ production (since day four) when compared with a low dose of HS1.

### 3.3. Viability of DCs and Production of Proinflammatory Cytokines

**Figure 3:**
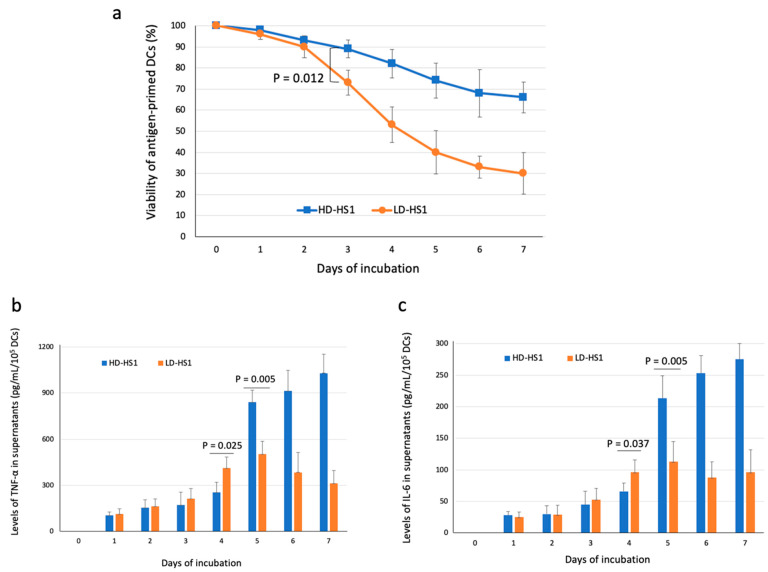
Kinetics of viability of DCs and production of proinflammatory cytokines in the in vitro DC-T cell model. (**a**) Viability of antigen-pulsed DCs in the incubation with CD8+ T cells (assessed by counting chamber with trypan blue dye exclusion); (**b**) TNF-α production in cell culture supernatants (assessed by ELISA); (**c**) IL-6 production in cell culture supernatants (assessed by ELISA). Data are presented as mean + SD among 10 subjects. Dose of individual antigens as following: LD-HS1 (low dose of S1 of SARS-CoV-2) as 110 ng/mL and HD-HS1 (high dose of S1 of SARS-CoV-2) as 550 ng/mL; *p*-values were determined by Wilcoxon signed-rank test.

Because antigen-presenting DCs themselves were also the targets of cytotoxicity of CD8+ T cells in this in vitro DC-T model [30], we assessed the viability of DCs in the wells of DC-T cell culture to evaluate the number of surviving APCs under the CTL activity of CD8+ T cells (i.e., the number of APCs still available to stimulate autologous CD8+ T cells in the cell cultures). We found the viability of LD-HS1-presenting DCs declined rapidly from day three of incubation with CD8+ T cells and was significantly lower than that of HD-HS1-presenting DCs (Figure 3a). The results were compatible with the higher levels of LD-HS1-specific cytotoxic activity of CD8+ T cells in the DC-T incubation.

Therefore, the higher viability of HD-HS1-presenting DCs may imply the lower HD-HS1-specific cytotoxic activity of CD8+ T cells and may lead to the persistence of HD-HS1-presenting DCs in the wells of DC-T cultures. We proposed these viable APCs may provide sustained stimulation for the co-incubated CD8+ T cells and then result in the marked lymphoproliferation and high levels of IFN-γ production from CD8+ T cells, which may cause subsequent overproduction of proinflammatory cytokines [31]. To test the hypothesis, we measured the levels of proinflammatory cytokines (TNF-α and IL-6) in the supernatants of incubation on different days of incubation. The levels of cytokine were presented as pg/mL/10^5^ DCs because the numbers of viable DCs were variable on different days of incubation. The kinetics showed an initially lower (at ≤ 4 days of incubation) but significantly higher levels of responses later (at ≥ 5 days of incubation) of TNF-α (Figure 3b) and IL-6 production (Figure 3c) by HD-HS1-presenting DCs when compared to those by LD-HS1-presenting DCs. Remarkable and persistently increased levels of TNF-α and IL-6 were noted from day five of incubation in response to HD-HS1-presenting DCs.

### 3.4. Dose-Related Responses between S1 Subunits and Perforin Expression

**Figure 4:**
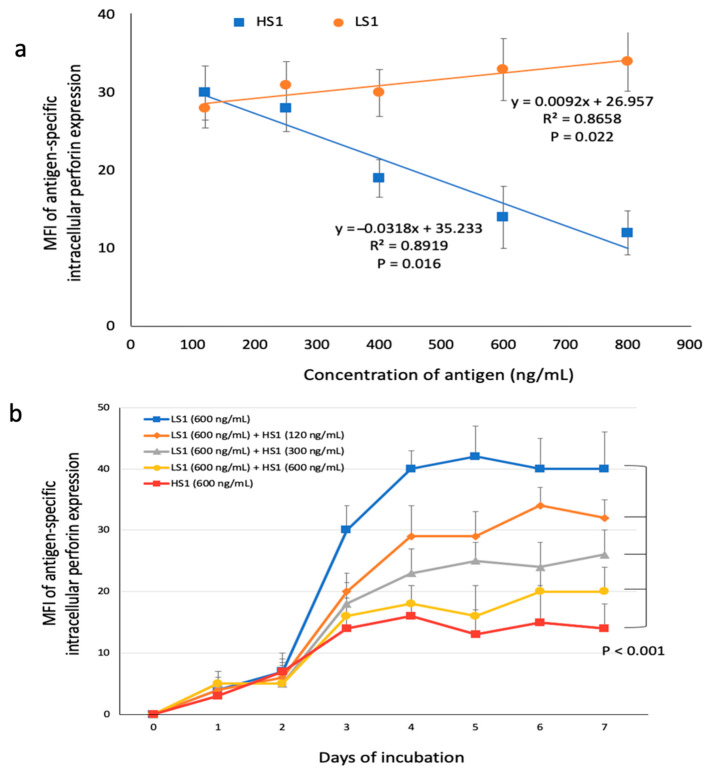
Suppression of intracellular perforin expression in CD8+ T cells by S1-presenting DCs. (**a**) Dose-related responses of mean fluorescence intensity (MFI) of antigen-specific intracellular perforin expression in CD8+ T cells were determined on day three of co-incubation with autologous DCs pulsed with variable doses of HS1 (S1 of SARS-CoV-2) or LS1 (S1 of HCoV-HKU1). Data are presented as mean + SD among 10 subjects. Linear correlation was evaluated using Pearson’s correlation coefficients. (**b**) Mixed S1 experiments to assess the impact of variable doses of HS1 on intracellular perforin expression in CD8+ T cells incubated with autologous DCs simultaneously pulsed with LS1 (600 ng/mL) and variable doses of HS1. Data are presented as mean + SD among 10 subjects. Statistical significance was determined using a repeated-measures one-way ANOVA.

To differentiate the insufficient expression of intracellular perforin expression in response to HD-HS1-presenting DCs was due to lower immunogenicity for CTL by HD-HS1 or due to the directly suppressive effects from HD-HS1-presenting DCs, we conducted an independent experiment to evaluate the impact of the different doses of S1 subunits on the intracellular perforin expression in CD8+ T cells. We pulsed immature DCs with varying doses of HS1 or LS1 in a range from 120 to 800 ng/mL and assessed the MFI of intracellular perforin expression in autologous CD8+ T cells that were incubated with the S1-presenting DCs on day three of co-incubation. We found that the levels of intracellular perforin expression in CD8+ T cells had a significant linear correlation with a negative slope if the CD8+ T cells co-incubated with HS1-presenting DCs, and a positive slope if the CD8+ T cells co-incubated with LS1-presenting DCs (Figure 4a).

To know whether the addition of HS1 could reduce the perforin expression in CD8+ T cells stimulated by LS1-presenting DCs, we performed the mixed S1 experiments to assess the levels of intracellular perforin expression of the CD8+ T cells that were incubated with autologous DCs pulsed simultaneously with LS1 (600 ng/mL) and varying doses of HS1 (Figure 4b). The results showed that the higher added doses of HS1 could result in the lower levels of intracellular perforin expression in CD8+ T cells induced by DCs, which were simultaneously pulsed with fixed doses of LS1. These data suggest that the lower intracellular perforin expression in CD8+ T cells in response to HD-HS1-presenting DCs may result from the directly suppressive effect of HS1 on perforin expression on CD8+ T cells in a dose-dependent manner, rather than the low immunogenicity of HS1.

### 3.5. The Suppressive Effect on Intracellular Perforin Expression on Different Subunits of Spike Glycoprotein

**Figure 5:**
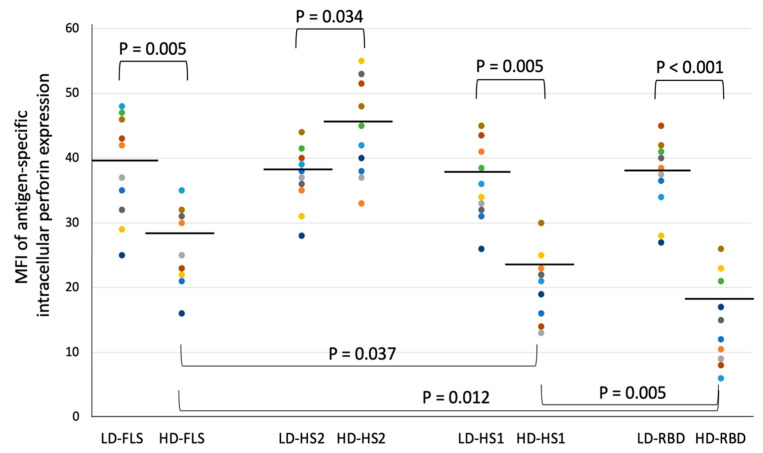
The antigen-specific intracellular perforin expression in CD8+ T cells in response to low dose (LD) or high dose (HD) FLS (full-length spike)-, HS1 (S1 of SARS-CoV-2)-, HS2 (S2 of SARS-CoV-2)-, or RBD (receptor-binding domain)-presenting DCs on day four of incubation in the in vitro DC-T model in 10 subjects. Horizontal lines indicate the data as mean values. The doses of antigens were calculated according to the relative molecular weights to achieve nearly equivalent amounts of protein molecules: LD-RBD as 40 ng/mL and HD-RBD as 200 ng/mL, LD-HS1 as 110 ng/mL and HD-HS1 as 550 ng/mL; LD-HS2 as 80 ng/mL and HD-HS2 as 400 ng/mL; LD-FLS as 200 ng/mL and HD-FLS as 1000 ng/mL; *p*-values were determined by Wilcoxon signed-rank test.

To know the impact of different subunits of spike glycoprotein from SARS-CoV-2 on inhibition of the intracellular perforin expression, we measured the intracellular perforin expression in CD8+ T cells in response to low dose or high dose of HS1-, HS2-, FLS-, or RBD-presenting DCs on day four of incubation (Figure 5). The data showed a high dose of FLS, HS1, and RBD, but not HS2, may have suppressive effects on intracellular perforin expression in CD8+ T cells. The ΔMFI of antigen-specific intracellular perforin expression in CD8+ T cells induced by low dose HS2-presenting DCs were significantly lower than those induced by high dose HS2-presenting DCs (Figure 5; 36.9 vs. 44.2, *p* = 0.034, by Wilcoxon signed-rank test). Discrepantly, the MFI of antigen-specific perforin expression in CD8+ T cells induced by high dose FLS- or HS1- or RBD-presenting DCs were significantly lower than those induced by low dose FLS- or HS1- or RBD-presenting DCs (25.8 vs. 38.4, *p* = 0.005; 20.5 vs. 36.0, *p* = 0.005; 14.7 vs. 36.9, *p* < 0.001, respectively, by Wilcoxon signed-rank test). In addition, the MFI of antigen-specific perforin expression induced by high dose RBD- presenting DCs were significantly lower than those induced by high dose FLS-presenting DCs or high dose HS1-presenting DCs (Figure 5). Furthermore, among the antigens or subunits of spike glycoprotein used in this experiment, HS2 is the only antigen that lacks the part of RBD. These data imply that RBD may play an essential role in the directly suppressive effect of spike glycoprotein from SARS-CoV-2 on intracellular perforin expression in CD8+ T cells.

## 4. Discussion

Multiple signaling pathways, cytokine cascades, and dysregulated cell activation may also be involved in the CSS [4,5,14,32]. To explain why high viral loads of SARS-CoV-2 exhaust CD8+ T cells and initiate the CSS [20,21], we showed the immunological characteristics of S1 subunit, especially RBD, of spike glycoprotein from SARS-CoV-2: the high dose of RBD-containing S1, when processed and presented by DCs, may aberrantly suppress or inhibit the intracellular perforin expression in CD8+ T cells, to significantly decrease the CTL capacity to kill spike-presenting cells (from day three of incubation); the persistence of spike-presenting cells may provide a sustained stimulation to CD8+ T cells and lead to a marked antigen-specific lymphoproliferation and IFN-γ production (from day four), and subsequent overproduction of proinflammatory cytokines such as TNF-α and IL-6 (from day five). That may help explain the immunological features of CSS and/or HLH in severe cases of COVID-19 that were similar to the clinical and pathological manifestation of severe cases of SARS and influenza [7,8,9,10,11,12,33]. Therefore, the overproduction of cytokines in this condition may actually reflect the insufficient cytotoxic activity of CD8+ T cells to kill virus-infected cells, rather than just the overactivity of immune responses.

Therefore, the data from our experiments of dose-related responses correlated with the previous clinical and laboratory observations that severe Covid-19 tends to have high viral loads, and disease progression correlates with functional impairment of cytotoxic lymphocytes [20,21]. We proposed the possibility that the perforin expression and specific cytotoxicity may be suppressed, and subsequent CSS may occur if SARS-CoV-2-infected patients are exposed to high quantities of spike glycoprotein, such as being infected by a high quantity of viruses or by highly replicated viral strains. The results were also compatible with the findings from a recently published mRNA vaccine trial. The higher dose of mRNA did not provide a stronger specific CD8+ T cell response [34]. Therefore, early initiation of effective antiviral treatment as possible, if available, to reduce the viral doses may be the key to reduce the risk of subsequent CSS.

Our study has some limitations. First, this study is very descriptive because we could not find a potential mechanism to explain how the spike proteins downregulate perforin expression. We plan to clarify the phenomenon by whole live SARS-CoV-2 virus, instead of purified protein, in our P3 laboratory in further studies. Second, we evaluated the immune responses in an in vitro culture environment by using purified recombinant viral antigens. The simplified in vitro DC-T model could control the dose of antigens and effector/target cell ratios and numbers but could not represent the in vivo conditions that may have complicated cell-to-cell interactions. Animal models or human studies are needed to test this hypothesis. Third, this study only studied the PBMCs from a small number of healthy volunteers aging from 25 to 48 years old. Thus, we do not know whether the results are reproducible if PBMCs were harvested from the subjects with older ages, with underlying diseases, or with immunosuppressive states. Fourth, we did not know the clinical relevance of the doses of spike glycoproteins we applied in this in vitro model. It is difficult to know how to correlate the dose of antigens in vitro with the virus load in a clinical setting of SARS-CoV-2 infection. Fifth, we did not demonstrate the CD83 or CD86 upregulation on DCs after pulsed with antigen in these experiments; thus, we could not really be sure of the maturation status of DCs. Sixth, intracellular IFN-γ staining that was not performed in this study would be a better way to determine the antigen-specific IFN-γ production from CD8+ T cells than IFN-γ production in supernatants.

The mechanism to explain why S1 subunit (especially RBD) may decrease the intracellular perforin expression remains vague. Several viral infections have been demonstrated to decrease the perforin expression and/or disable perforin function of CD8+ T cells, such as Epstein-Barr virus (EBV) and avian influenza (H5N1) virus, through the abnormalities in perforin structure and inhibition of perforin activation disabling the molecule from binding to target cell membrane [12,35]. In a future study, to determine the granzyme levels and/or to assess mRNA levels of perforin and/or to investigate the possible role of miRNA may help elucidate the possible mechanism.

In conclusion, through the data from this in vitro DC-T model, we hypothesize that the receptor-binding domain (RBD)-containing S1 subunit of spike glycoprotein from SARS-CoV-2 may suppress the intracellular perforin expression in CD8+ T cells to decrease the cytotoxic capacity to kill spike-presenting cells in a dose-dependent manner, and the persistence of antigen presentation may result in the overactivity of CD8+ T cells and may initiate the subsequent hyperproduction of proinflammatory cytokines. Therefore, CSS may actually reflect the insufficient CD8+ CTL in the face of the high quantities of viruses or highly replicated strains of SARS-CoV-2. The results may have implications on dose selection for the development of vaccines that apply recombinant spike proteins [36].
